# Evolutionary ecology of pipefish brooding structures: embryo survival and growth do not improve with a pouch

**DOI:** 10.1002/ece3.2139

**Published:** 2016-04-24

**Authors:** Ines Braga Goncalves, Ingrid Ahnesjö, Charlotta Kvarnemo

**Affiliations:** ^1^ Department of Evolutionary Biology and Environmental Studies, Animal Behaviour University of Zurich Winterthurerstrasse 190 8057 Zurich Switzerland; ^2^ Department of Biological and Environmental Sciences University of Gothenburg PO Box 463 40530 Gothenburg Sweden; ^3^ Department of Ecology and Genetics/Animal Ecology Uppsala University Norbyvägen 18D 75236 Uppsala Sweden

**Keywords:** Egg size, embryo growth, embryo survival, environmental conditions, low oxygen, parental care, paternal care, Syngnathidae

## Abstract

For animals that reproduce in water, many adaptations in life‐history traits such as egg size, parental care, and behaviors that relate to embryo oxygenation are still poorly understood. In pipefishes, seahorses and seadragons, males care for the embryos either in some sort of brood pouch, or attached ventrally to the skin on their belly or tail. Typically, egg size is larger in the brood pouch group and it has been suggested that oxygen supplied via the pouch buffers the developing embryos against hypoxia and as such is an adaptation that has facilitated the evolution of larger eggs. Here, using four pipefish species, we tested whether the presence or absence of brood pouch relates to how male behavior, embryo size, and survival are affected by hypoxia, with normoxia as control. Two of our studied species *Entelurus aequoreus* and *Nerophis ophidion* (both having small eggs) have simple ventral attachment of eggs onto the male trunk, and the other two, *Syngnathus typhle* (large eggs) and *S. rostellatus* (small eggs), have fully enclosed brood pouches on the tail. Under hypoxia, all species showed lower embryo survival, while species with brood pouches suffered greater embryo mortality compared to pouchless species, irrespective of oxygen treatment. Behaviorally, species without pouches spent more time closer to the surface, possibly to improve oxygenation. Overall, we found no significant benefits of brood pouches in terms of embryo survival and size under hypoxia. Instead, our results suggest negative effects of large egg size, despite the protection of brood pouches.

## Introduction

One striking difference between aquatically and terrestrially reproducing animals is the size of eggs produced in each environment. Apart from rare exceptions (e.g., shark eggs), eggs of aquatic animals are not nearly as large as those of birds or reptiles. Aquatic environments differ from terrestrial ones in multiple ways, including in having lower and more variable levels of dissolved oxygen (Keister et al. 2000; Nilsson and Östlund‐Nilsson 2008). Thus, low oxygen availability has long been assumed to constrain the evolution of egg size of aquatic organisms, particularly of fish (van den Berghe and Gross 1989; Quinn et al. 1995; Kolm and Ahnesjö 2005). A developing embryo acquires oxygen through the egg membrane and the perivitelline fluid (Jones 1986; Strathmann and Strathmann 1995). Primarily, this process is based on diffusion and both the egg membrane and the perivitelline fluid act as barriers to embryo oxygenation.

Fish eggs are usually spherical, a shape that has an unfavorable surface area to volume ratio, with an increase in egg size resulting in a greater increase of volume compared to the surface area. Embryo oxygen consumption (i.e., respiration) is commonly assumed to be proportional to egg volume (but see Einum et al. 2002), whereas oxygen availability is related to the surface area of the egg (Hendry and Day 2003; Kolm and Ahnesjö 2005). Thus, embryos from larger eggs are assumed to have greater difficulties acquiring the oxygen required for successful development compared to embryos from smaller eggs (van den Berghe and Gross 1989; Quinn et al. 1995; Kolm and Ahnesjö 2005; Bakker et al. 2006). However, so far, little evidence of such patterns has been found. Instead, an increasing number of studies have questioned this assumption (Einum et al. 2002; Rombough 2007; Braga Goncalves et al. 2015b).

Anthropogenic impacts affect literally all marine ecosystems; with seagrass meadows being some of the most affected areas (Halpern et al. 2008). Furthermore, oxygen solubility in water decreases with increasing temperatures, which occur most frequently in shallow coastal areas (Benson and Krause 1980; Sherwood et al. 1992; Keeling and Garcia 2002) leading to hypoxia. Increased levels of hypoxia in estuaries and shallow areas (Diaz and Rosenberg 2008) that are essential for the reproduction of many aquatic organisms have led to a renewed interest in egg size evolution, embryo development, and parental care in aquatic systems. Low ambient oxygen conditions have detrimental effects on embryos, including reduced metabolic efficiency leading to slower developmental rates, delayed hatching, and increased mortality (e.g., Alderdice et al. 1958; Bradford and Seymour 1985; Kamler 1992; Mills and Barnhart 1999).

Dissolved oxygen saturation in aquatic environments is affected by many ecological factors (such as salinity, temperature, current velocities, rainfall, and algae), and species can vary in their sensitivity and tolerance to hypoxia (Nilsson and Östlund‐Nilsson 2008). Consequently, the detection of general patterns in constraints and adaptations to hypoxia across species can be difficult to assess. Studies that compare populations, within species, that experience different ecological conditions and studies that compare closely related species that experience similar ecological conditions can, thus, shed important light on how egg size, complexity of parental care, and oxygen availability interact to affect offspring development in aquatic environments.

Broadcast spawning species usually produce small and numerous eggs, while species that provide some form of care tend to produce fewer and larger eggs (Shine 1978; Gross and Sargent 1985; Sargent et al. 1987; Kolm and Ahnesjö 2005). The evolution of egg size and parental care in aquatic environments is still, despite decades of research efforts, a contentious issue. Theoretical models tend to predict that parental care is favoured when adult mortality is high and development slow, and selection favors increased time spent in the relatively safer life‐history stage, in this case, the egg (e.g., Shine 1978; Sargent et al. 1987; Klug and Bonsall 2010). Once parental care evolves, coevolution of parental care (more or longer care) and egg size (selection for larger sizes) is predicted to occur (Nussbaum and Schultz 1989; Kolm and Ahnesjö 2005). In many fish species, parental care includes protection of the embryos from predators, prevention of diseases by cleaning the eggs and improved access of oxygen to embryos through fanning, brooding or other special constructions. For example, common goby (*Pomatoschistus microps*) and sand goby (*P. minutus*) males build wider nest openings under hypoxic conditions (Jones and Reynolds 1999b; Lissåker and Kvarnemo 2006). Also, longfinned gobies (*Valenciennea longipinnis*) and hornyhead chubs (*Nocomis biguttatus*) prefer to build their nests in places where oxygen‐rich water runs through the nests (Takegaki 2001; Wisenden et al. 2009). Furthermore, common, sand, and longfinned goby males increase their fanning frequency and duration under hypoxia (Jones and Reynolds 1999a; Takegaki and Nakazono 1999; Lindström et al. 2006; Lissåker and Kvarnemo 2006). In at least some syngnathids (e.g., *Syngnathus typhle, S. floridae, S. fuscus, S. abaster, Hippocampus hippocampus*), males transfer oxygen to the embryos through the brood pouch (Berglund et al. 1986b; Carcupino et al. 2002; Stölting and Wilson 2007; Ripley 2009; Braga Goncalves et al. 2015a,b). Given these observations, it has been proposed that parental care in fishes may have evolved, at least partially, to protect developing embryos from hypoxia (Kolm and Ahnesjö 2005), possibly as a consequence of selection of larger egg sizes.

The family Syngnathidae, pipefishes, seahorses, and seadragons, is characterized by extensive and highly specialized paternal care that ensures full certainty of paternity and presents a wide diversity in brooding structures at the genus level (Wilson et al. 2001; Coleman and Jones 2011). While in all species males brood the eggs on their bodies throughout the developmental period, syngnathids vary greatly in extent and form of brood care (Carcupino et al. 2002; Wilson et al. 2003). Brood pouch complexity ranges from a complete lack of pouch, in which the eggs are attached to the skin of the male's tail or abdomen, through partially enclosed pouches consisting of lateral pouch plates, to fully enclosed pouches formed by two skin folds that seal in the middle during brooding, and the sac‐like fully closed pouch of seahorses (Herald 1959; Dawson 1985; Wilson et al. 2001). These different types of brood pouches have traditionally been thought to have evolved due to benefits of improved reproductive success via offspring survival (Wilson et al. 2001). For example, in several pipefish species with brood pouches, males are able to provide nutrients to the developing embryos (Haresign and Shumway 1981; Berglund et al. 1986b; Ripley and Foran 2006; Ripley 2009; Kvarnemo et al. 2011). Furthermore, species with enclosed brood pouches (e.g., *Syngnathus* spp. and seahorses) are able to osmoregulate and oxygenate the offspring during brooding (Quast and Howe 1980; Haresign and Shumway 1981; Berglund et al. 1986b; Carcupino et al. 1997, 2002; Partridge et al. 2007; Stölting and Wilson 2007; Ripley 2009; Ripley and Foran 2009; Braga Goncalves et al. 2015a) and preliminary evidence suggests that species with more complex brood pouches have larger eggs (Braga Goncalves et al. 2011).

**Figure 1:**
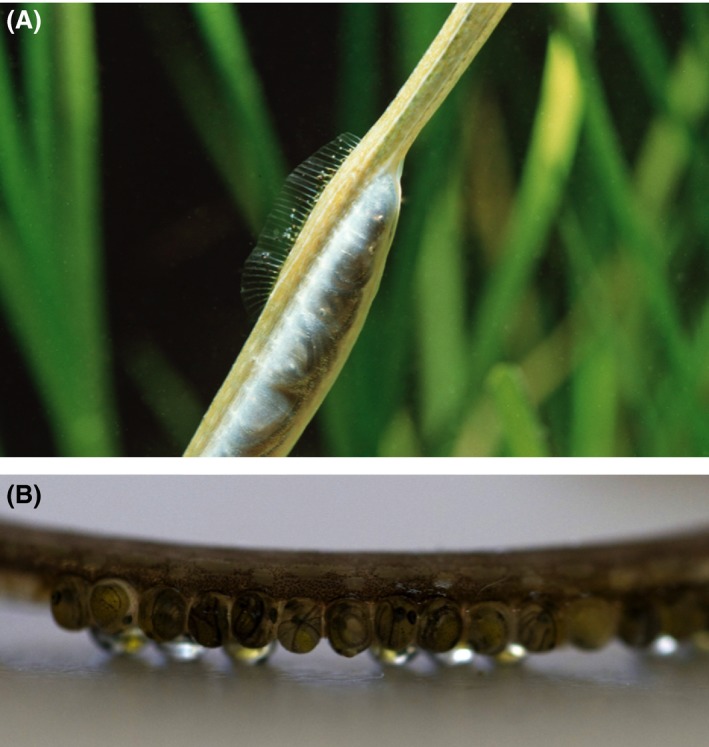
Close‐up images of (A) the brood pouch of a *S. typhle* male during the last stages of pregnancy (photo credit: Ola Jennersten) and (B) the ventral side of a brooding *N. ophidion* male, where the embryos are clearly visible (photo credit: Josefin Sundin).

In this study, we focus on four temperate pipefish species that share the same habitat and ecological conditions during the breeding season: two pipefish species with fully enclosed brood pouches (*Syngnathus rostellatus* and *S. typhle*, Fig. 1A) and two species without pouches (*Nerophis ophidion* and *Entelurus aequoreus*, Fig. 1B). Experimentally, we assess how embryo size, embryo survival, and male behavior are affected by (1) hypoxia or normoxia, (2) egg size, and (3) type of brooding structure, allowing us to investigate how these factors interact. We predicted that *N. ophidion* and *E. aequoreus* males that brood embryos exposed to the ambient water would show behavioral adaptations to improve oxygenation to their embryos. Furthermore, we predicted that embryos of the two species that have brood pouches (*S. rostellatus* and *S. typhle*) would be less affected by hypoxic conditions, if the pouch has the buffering or oxygen provisioning function it has been suggested to have. Regarding egg size, we predicted that species brooding small eggs would be less affected by hypoxia, showing fewer detrimental effects in terms of embryo length, weight, and survival.

## Materials and Methods

### Ethics statement

This study was carried out according to Swedish law, with an ethical approval provided by the Swedish Animal Welfare Agency (permit no. 11‐2007). This study did not involve any endangered or protected species.

### Study species and site

The west coast of Sweden is inhabited by six pipefish species. Four of these species (*S. rostellatus, S. typhle, N. ophidion, and E. aequoreus*) are found in similar coastal seagrass habitats, in good numbers, and are possible to mate and house well at the field stations. These four species have about the same breeding season and experience similar ecological conditions, although larger individuals tend to occur in slightly deeper water (Vincent et al. 1995).

Nilsson's pipefish (*S. rostellatus*) and the broad‐nosed pipefish (*S. typhle)* are closely related species (Dawson 1985) that differ most notably in size, with *S. rostellatus* being the smaller of the two (Table S1). Both species have a brood pouch on their tail, formed by two skin folds that merge and form a closed brood pouch after mating. Both species are polygynandrous (Vincent et al. 1995) and males usually brood eggs from multiple females, being restricted only by the number of eggs that can be fitted into the brood pouch (Berglund et al. 1988, 1989; Jones et al. 1999; Rispoli and Wilson 2008). Females mature eggs continuously and tend to transfer eggs to different males throughout the breeding season (Berglund et al. 1989; Jones et al. 1999; Sogabe and Ahnesjö 2011). The sexes of *Syngnathus* sp. can be identified relatively easily all year round due to sex‐specific differences in relative lengths of trunk and tail (Winkler et al. 2012) and are especially easy to tell apart during breeding season through the presence of the brood pouch in males.

The straight‐nosed pipefish (*N. ophidion*) and the snake pipefish (*E. aequoreus*) also differ significantly in body size, with the snake pipefish being one of the longest pipefish species (Table S1). In both species, the females are larger and more ornamented than the males (Berglund et al. 1986a; Rosenqvist 1990). Males receive eggs from a single female at a time and consistent with this, females mature eggs in synchronous batches, and transfer all the mature eggs to one male at a time (Berglund et al. 1989; Sogabe and Ahnesjö 2011). Both species lack a brood pouch and embryos are brooded on the ventral surface of the male trunk, in small epidermal depressions that are formed after the eggs are transferred.

During the breeding season, these species migrate from deeper waters into shallow bays dominated by sand, eelgrass (*Zostera marina*), and patches of brown algae (*Fucus* and *Laminaria* spp). Importantly, higher temperatures and weak currents in the summer commonly lead to algal overgrowth on the seagrass meadows inhabited by our study species (Pihl et al. 1999) causing the water to be hyperoxic during the day due to photosynthesis and hypoxic at night due to respiration (Greve et al. 2003; Moore 2004; Moore and Jarvis 2008). All species included in this study are inactive, spending most of the time either lying on the bottom or aligning vertically with the eelgrass and brown algae in their habitat, making them very cryptic (Vincent et al. 1995).

The study was carried out on the Swedish west coast at the Sven Lovén Centre for Marine Sciences, Kristineberg (58°15′N, 11°28′E) between May and July 2007 and 2008. Animals were collected from eelgrass (*Zostera marina*) meadows using a beam trawl pulled behind a boat. Fish were brought into the laboratory and kept in large storage barrels (225 L), separated by species and sex (all species), and by size (*S. typhle* females only). Both barrels and experimental aquaria were equipped with artificial eelgrass, a flow‐through system of continuously renewed natural seawater providing natural salinity conditions, and a light cycle of 16: 8 L: D hours per day. Water temperature was kept constant at 14–15°C throughout the study. Fish were fed three times per day with *Artemia salina* (all pipefish species), supplemented with wild caught crustaceans (*Praunus flexuosa*,* Crangon crangon,* and copepods) for *S. typhle* and *E. aequoreus*. Storage barrels were cleaned daily, mating aquaria every second day, and brooding aquaria every third day.

### Experimental design

#### Matings

Matings for *S. rostellatus*,* S. typhle,* and *N. ophidion* were performed by introducing groups of males and females in 140–160 L mating aquaria. For the largest species, *E. aequoreus,* matings were performed in 225‐L barrels or in a 3 m^3^‐large aquarium at the nearby Klubban Biological Station. The standard body lengths (SL) of all fish were taken to the nearest mm. Females were measured before being introduced into the mating tanks, whereas males were measured after mating, before they were moved to the brooding aquaria. Male and female sizes are given in Table S1. For *S. rostellatus*,* E. aequoreus,* and *N. ophidion,* a range of female lengths was used, reflecting size distributions in the field. However, for *S. typhle* similar sized males were introduced in the mating aquaria either with groups of larger or of smaller females, which produce large and small eggs, respectively (Braga Goncalves et al. 2011), as part of another study (Braga Goncalves et al. 2015b). Hence, for this species, two separate groups are analyzed: one group of males brooding embryos from small females (*S. typhle* s) and another group of males brooding embryos from large females (*S. typhle* l). It is important to note that the males in these two groups did not differ in body size (Braga Goncalves et al. 2015b), only in the average size of the eggs they brooded. In addition, as will be explained in the section on statistical analyses, we classified species according to whether their average egg size was larger or smaller than 1.5 mm in diameter. This division was based on a natural gap in egg size between the species found in a previous study of the same populations of pipefishes as in this study (see Fig. 1 in Braga Goncalves et al. 2011). A few details are worth pointing out: First, in the current between‐species comparison, both experimental groups of *S. typhle* are classified as having large eggs. Secondly, *S. rostellatus* males were mated with females of a range of sizes and, although egg size is positively and significantly correlated to female size, all eggs for this species were well below our 1.5 mm threshold. Thirdly, in *N. ophidion* and *E. aequoreus*, females of all body sizes produce eggs that are of similarly small size; thus, egg size is not correlated to female size.

Because large *S. typhle* females produce enough eggs to fill up almost three males of similar size during the course of one pregnancy, and small females can only fill about one male of similar size during the same time period (Berglund and Rosenqvist 1990; Ahnesjö 1995), females and males were put in mating aquaria in ratios of 1: 2 for large females (four females: eight males) and 1: 1 for small females (eight females: eight males, see Braga Goncalves et al. 2015b for more details). These ratios ensured that males mated and filled up their brood pouches quickly. For the remaining species, the following ratios of individuals were used, per mating aquarium: *S. rostellatus*: 2: 1 (twenty females: ten males), *N. ophidon*: 3: 1 (twelve females: four males), *E. aequoreus*: 2: 1 (four females: two males). Females were replaced with new ones if no mating occurred within 3 days.

Males were kept in the mating aquaria until they had mated (*N. ophidion* and *E. aequoreus*) or until their pouches were filled with eggs (*Syngnathus* spp). One day thereafter, males were measured and individually identified before being moved into brooding aquaria. We took pictures (Canon IXUS 850 IS) of the ventral side of all *N. ophidion* and *E. aequoreus* males to record egg numbers (Fig. S1).

#### Oxygen treatments

Within each species group, mated males were transferred to separate brooding aquaria (L × W × H: 26 × 45 × 40 cm or 26 × 35 × 35 cm) and each tank housed one male of each species. Each brooding aquarium was then allocated at random to an oxygen treatment and was provided either fully oxygenated water (normoxic treatment: 100% O_2_ saturation) or hypoxic water (hypoxic treatment: 40% O_2_ saturation). Water O_2_ saturation in the hypoxic treatment was reduced using a MiniModule 1.7* 5.5 Membrane Contactor (Liqui‐Cel, Celgard, Inc, Charlotte, NC), which flowed nitrogen in a counter‐current system in relation to the direction of the water flow, continuously replacing oxygen with nitrogen in incoming water. This way all experimental aquaria had flowing seawater. Normoxic aquaria were equipped with air stones to maintain high O_2_ saturation. Oxygen levels in all aquaria were monitored daily with a portable oxygen meter (Handy Delta, OxyGuard, Tekno Trading AB, Säffle, Sweden) and in the hypoxia‐treated tanks the nitrogen flow was adjusted when needed.

The treatment period was set to 18 days to ensure that embryos had developed eyespots. Eighteen days amounts to just less than half of the total brooding period of, at least, *S. typhle* and *N. ophidion* at 14–15°C (Berglund et al. 1986b; Ahnesjö 1995). At experimental termination, males were sacrificed by placing them in 1 mL 2‐phenoxyethanol/liter seawater for 5 min, followed by severing the spinal column posterior to the operculum. Males were preserved in 70% ethanol in individual vials for later analysis of embryo survival and size. All females were released back close to the site of collection after the matings.

### Data collection

#### Embryo survival

In the species that have enclosed brood pouches (*S. typhle* and *S. rostellatus*), for each male, we first removed all embryos from the brood pouch. Relative embryo survival was assessed by dividing the number of developing embryos by the total number of eggs in the brood pouch. In species that do not have brood pouches (*N. ophidion* and *E. aequoreus*), initial number of eggs received by each male was counted from the photographs taken after mating (see Matings section and Fig. S1). Embryos were removed from the trunk of each male, and relative embryo survival was calculated by dividing the number of developing embryos by the initial number of eggs.

#### Embryo size

Between three and five embryos from each male were separated from the egg membrane and yolk sac. Individual pictures of the embryos were taken using a camera (Leica DFC420 A) attached to a stereomicroscope (Leica MZ16 A). The total length (tip of rostrum to tip of tail) of each embryo (± 0.01 mm) was measured from the photographs using the program Leica Application Suite, version 2.7.0.RI (Build: 1294). In addition, ten embryos were separated from their egg membrane and yolk sac, dried in a heating cupboard (60°C) for 1 week and weighed twice on a Sartorius LE26P microbalance (± 2 *μ*g). We calculated the average embryo length and dry weight for each brood to obtain estimates for each male at the end of the 18‐day brooding period.

#### Ventilation rates and male behavior

On days 1, 9, and 18 of brooding, ventilation rates, and 10‐min video recordings were conducted on each male. Ventilation rates (number of opercular movements per minute) were collected through direct observation of each individual for a period of 30 sec. Videos were analyzed using JWatcher v1.0. (http://galliform.psy.mq.edu.au/jwatcher/) From the videos, proportion of time spent in the top half of the water column and proportion of time spent swimming were analyzed for each male at each occasion.

### Statistical analysis

All data were analyzed using SPSS17 (SPSS Inc. Chicago, IL, USA) for Windows and PERMANOVA+ for PRIMER v6 (PRIMER‐E, Plymouth, UK). PERMANOVA+ was chosen because it relies on permutations to calculate data distributions. *S. typhle* males were analyzed as two separate groups of males, brooding either small (*S. typhle* s) or large eggs (*S. typhle* l), but note that the groups did not differ in male body length (see Braga Goncalves et al. 2015b). The presence of outliers was tested using Grubb's test for outliers (ESD method), and as a result, two *N. ophidion* and one *S. typhle* males were removed from the analyses, due to having a Z‐ratio higher than the critical Z‐ratio (all *P* < 0.05). All eggs in these males were unfertilized.

First, we used the procedure Permdisp in the add‐on PERMANOVA+ for Primer to assess whether differences between groups were due to differences in dispersion of the datasets. As all groups had similar dispersion, we used Permanova to assess: (1) the effects of group (fixed factor (FF): *S. typhle* l, *S. typhle* s, *S. rostellatus*,* E. aequoreus,* and *N. ophidion*) of oxygen treatment (FF: 100% and 40% O_2_) and of their interaction on overall relative embryo survival, embryo length, and dry weight and (2) the effects of group, oxygen treatment, day [FF: days 1, 9 and 18, repeated measure (RM)], and male ID (random factor (RF) nested within oxygen treatment and species group: 141 individuals), on overall ventilation rates, proportion of time spent swimming and proportion of time spent in the upper part of the aquarium. In addition, we assessed how oxygen treatment interacted with (3) brood pouch presence (FF: 2 levels, no pouch: *N. ophidion* and *E. aequoreus*; pouch: *S. typhle* l, *S. typhle* s, *S. rostellatus*) and d) egg size (FF: 2 levels, large eggs (>1.5 mm in diameter): *S. typhle* l and *S. typhle* s, small eggs (<1.5 mm): *S. rostellatus*,* N. ophidion* and *E. aequoreus*), to affect embryo survival, length, and dry weight. Using the averages of the three recordings for each male, that is, no repeated measure, we also tested for influences of brood pouch presence on male brooding behavior and ventilation rates.

Relative embryo survival was arcsine‐transformed, embryo length, and dry weight were square‐root‐transformed, and proportions of time spent in the upper half of the water column and swimming were arcsine‐square‐root‐transformed. Furthermore, we normalized all response variables (each variable had its mean subtracted and was divided by its standard deviation) to achieve a common scale between the variables before producing a resemblance matrix based on Euclidean distances (Clarke and Gorley 2006; Anderson et al. 2008). Interaction terms with p (perm)‐values > 0.2 were sequentially removed from the models (final models of main analyses are presented in Tables 1 and 2), starting with highest degree interactions, followed by within level least significance. To reduce multiple testing and the associated probability of type I errors, permutational ANOVAs were performed on the separate response variables only if the permutational MANOVA revealed significant effects. The following options were chosen for all analyses: type III sums of squares, fixed effects sum to zero, model: permutation of residuals under a reduced model, number of permutations: 9999. Significance level for all tests was set at *P* < 0.05.

**Table 1:** Permutational ANOVA analyses showing the effects of group (*S. typhle* l, *S. typhle* s, *S. rostellatus, N. ophidion,* and *E. aequoreus*), oxygen treatment (100% vs. 40% O_2_ saturation), and their interaction on relative embryo survival (%), average embryo length, (mm) and average embryo dry weight (*μ*g). *N* = 141. Analyses were performed on transformed variables

Source	Embryo survival				Average embryo length				Average embryo weight			
	df	MS	Pseudo‐*F*	p (perm)	df	MS	Pseudo‐*F*	P (perm)	df	MS	Pseudo‐*F*	p (perm)
Group	4	3.73	4.42	0.002	4	22.85	96.45	<0.001	4	8.30	11.30	<0.001
Oxygen level	1	6.88	8.16	0.005	1	8.44	35.60	<0.001	1	7.90	10.77	0.001
Oxygen* grouvp	4	2.10	2.49	0.047	4	1.75	7.38	<0.001	4	1.28	1.75	0.139
Residual	131	0.84			131	0.24			131	0.73		

**Table 2:** Permutational ANOVA analyses showing the effects of group (*S. typhle* l, *S. typhle* s, *S. rostellatus*,* N. ophidion,* and *E. aequoreus*), oxygen treatment (100% vs. 40% O_2_ saturation), day (repeated measure: days 1, 9, and 18), and male ID, on proportion of time spent swimming, proportion of time spent on the upper half of the water column and ventilation rates. *N* = 131. Analyses were performed on transformed variables

Source	Time spent swimming				Time spent in upper half of water column				Ventilation rates			
	df	MS	Pseudo‐*F*	p (perm)	df	MS	Pseudo‐*F*	p (perm)	df	MS	Pseudo‐*F*	p (perm)
Group	4	52.88	94.60	<0.001^a^	4	12.55	13.04	<0.001^a^	4	39.69	76.35	<0.001^a^
Oxygen treatment	1	0.62	1.11	0.286^a^	1	8.09	8.40	0.005^a^	1	79.01	152.01	<0.001^a^
Day	2	0.82	1.96	0.145^a^	2	8.87	11.83	<0.001^a^	2	7.33	27.54	<0.001^a^
Male ID	121	0.56	1.33	0.027^a^	121	0.96	1.28	0.054^a^	121	0.52	1.95	<0.001^a^
Residual	264	0.42^b^			264	0.75^b^			264	0.27^b^		

a Term mean square was tested against pooled mean square of residuals and all interaction terms.

b Mean square of pooled residuals and interaction terms.

## Results

### Embryo survival and embryo size

**Figure 2:**
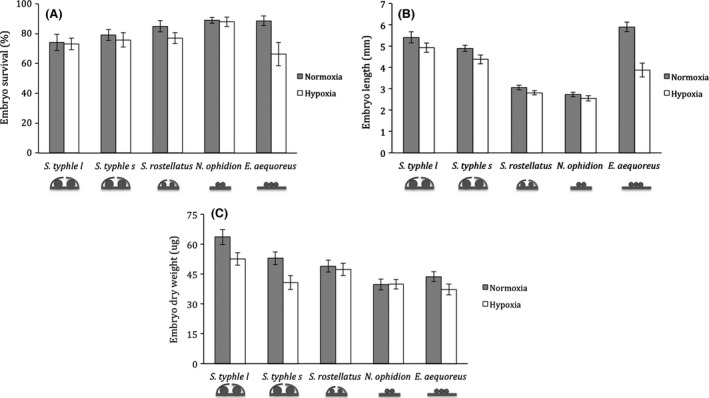
Average ± SE (A) embryo survival (%), (B) embryo length (mm), and (C) embryo dry weight (*μ*g) in normoxia (100% O_2_ saturation) and hypoxia (40% O_2_ saturation) by *S. typhle* males brooding large (*S. typhle* l, *N* = 22) or small (*S. typhle* s, *N* = 24) eggs, *S. rostellatus* (*N* = 32), *N. ophidion* (*N* = 37), and *E. aequoreus* (*N* = 26) males. At the bottom of the figures are shown schematic drawings displaying the presence or absence of brood pouch (as seen in cross section) and egg size category for each group.

We found clear effects of oxygen treatment (permutational MANOVA: pseudo‐*F*_1,131_ = 12.8, p (perm) < 0.001), group (permutational MANOVA: pseudo‐*F*_4,131_ = 19.23, p (perm) < 0.001), and of their interaction (permutational MANOVA: pseudo‐*F*_4,131_ = 2.83, p (perm) = 0.003) on overall embryo survival and embryo size, showing that groups were affected differently by our two oxygen conditions. Separately, hypoxia had a consistent significant negative effect on all variables and embryo survival, length, and weight differed significantly between groups (Table 1, Fig. 2). Furthermore, we found a significant interaction between oxygen treatment and group (Table 1) with *E. aequoreus* showing a more pronounced negative effect of hypoxia on embryo survival (Fig. 2A) and on embryo length (Fig. 2B), but not on embryo dry weight, compared to the other groups (Fig. 2C).

The brood pouch group had significantly lower embryo survival than the pouchless group (permutational ANOVA: pseudo‐*F*_1,137_ = 9.02, p (perm) = 0.003). The negative effect of hypoxia (oxygen treatment) was significant and similar across groups, that is, the interaction between the factors was not significant (permutational ANOVA: O_2_ *treatment*: pseudo‐*F*_1,137_ = 6.11, p (perm) = 0.013, O_2_ *treatment* * *pouch*: pseudo‐*F*_1,137_ = 0.01, p (perm) = 0.92). We also found a significant effect of egg size category on average embryo survival, with the large egg size group (the two *S. typhle* groups) showing significantly lower embryo survival compared to the small egg group (*S. rostellatus*,* N. ophidion,* and *E. aequoreus*: permutational ANOVA: *egg size*: pseudo‐*F*_1,137_ = 10.46, p (perm) = 0.001). Egg size category and oxygen treatment did not interact significantly (O_2_ *treatment* * *egg size*: pseudo‐*F*_1,137_ = 0.40, p (perm) = 0.53).

The presence of brood pouch had a significant positive effect on embryo length (permutational ANOVA: pseudo‐*F*_1,137_ = 6.53, p (perm) = 0.011). Yet, average embryo length in both pouch categories was similarly negatively affected by low‐oxygen treatment (permutational ANOVA: O_2_ *treatment*: pseudo‐*F*_1,137_ = 11.89, p (perm) < 0.001, O_2_ *treatment* * *pouch*: pseudo‐*F*_1,137_ = 2.49, p (perm) = 0.12). The large egg size category (*S. typhle* l and *S. typhle* s) had significantly longer embryos than those in the small egg size category (permutational ANOVA: pseudo‐*F*_1,137_ = 65.33, p (perm) < 0.001). Yet, both egg size categories were negatively affected by oxygen treatment (permutational ANOVA: pseudo‐*F*_1,137_ = 11.19, p (perm) < 0.001) and the interaction was nonsignificant (permutational ANOVA: O_2_ *treatment* * *egg size*: pseudo‐*F*_1,137_ = 0.68, p (perm) = 0.42). Similar results were found for average embryo dry weight, with the group with brood pouches having significantly heavier embryos than the pouchless group (permutational ANOVA: *pouch*: pseudo‐*F*_1,137_ = 28.01, p (perm) < 0.001, O_2_ *treatment*: pseudo‐*F*_1,137_ = 6.84, p (perm) = 0.008, O_2_ *treatment* * *pouch*: pseudo‐*F*_1,137_ = 1.31, p (perm) = 0.26), and the larger egg size category had significantly heavier embryos compared to the small egg size category (permutational ANOVA: *egg size*: pseudo‐*F*_1,137_ = 18.74, p (perm) < 0.001, O_2_: pseudo‐*F*_1,137_ = 10.72, p (perm) < 0.001, O_2_ * *egg size*: pseudo‐*F*_1,137_ = 4.32, p (perm) = 0.04). The significant interaction effect between oxygen treatment and egg size category shows that, in low‐oxygen conditions, embryos of large eggs (*S. typhle* l and s) suffered significantly greater reductions in weight compared to embryos of species with small eggs (Fig. 2C).

### Male behavior

We found significant individual variation in overall response to the oxygen treatments (permutational MANOVA: pseudo‐*F*_121,264_ = 1.42, p (perm) < 0.001). Still, there were clear effects of group (permutational MANOVA: pseudo‐*F*_4,264_ = 51.50, p (perm) < 0.001), oxygen treatment (permutational MANOVA: pseudo‐*F*_1,264_ = 42.97, p (perm) < 0.001), and day of brooding (permutational MANOVA: pseudo‐*F*_2,264_ = 17.02, p (perm) < 0.001) on brooding male ventilation rates and activity patterns. These effects were equally strong when we analyzed the behavioral variables separately, with the exception of proportion of time spent swimming, which was unaffected by both oxygen treatment and day of brooding (Table 2). Hypoxia increased the proportion of time spent in the upper half of the water column and ventilation rates.

**Figure 3:**
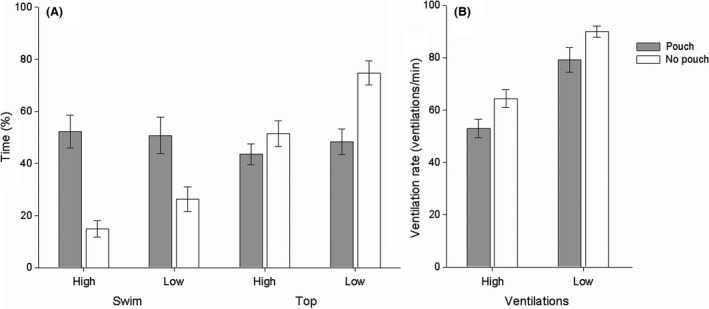
Average ± SE (A) time spent swimming (%), time spent on the upper half of the water column (%) and (B) ventilation rates, of males of species with brood pouches (gray, *S. typhle* s, *N* = 24, *S. typhle* l, *N* = 22, and *S. rostellatus*,* N* = 32) and without brood pouches (white, *N. ophidion*,* N* = 37 and *E. aequoreus*,* N* = 26), and brooding in normoxia (high) or in hypoxia (low). Measurements are based on averages of the three recordings performed on days 1, 9, and 18 of the experimental brooding period.

The brood pouch group had significantly lower ventilation rates (2‐way permutational ANOVA: pseudo‐*F*_1,127_ = 9.44, p (perm) = 0.002), spent significantly more time swimming (2‐way permutational ANOVA: pseudo‐*F*_1,127_ = 28.23, p (perm) < 0.001), and less time close to the water surface (2‐way permutational ANOVA: pseudo‐*F*_1,127_ = 11.87, p (perm) = 0.001), irrespective of oxygen treatment (Fig. 2). Average ventilation rate and proportion of time spent in the upper half of the water column, but not swimming activity, were significantly affected by oxygen treatment (2‐way permutational ANOVA: ventilation rate: pseudo‐*F*_1,127_ = 49.01, p (perm) < 0.001; proportion of time spent swimming: pseudo‐*F*_1,127_ = 0.33, p (perm) = 0.57; proportion of time in upper half of water column: pseudo‐*F*_1,127_ = 10.62, p (perm) = 0.001). Moreover, under hypoxia, individuals in the “pouchless” species spent significantly more time close to the water surface compared to individuals belonging to the pouch category (2‐way permutational ANOVA: pseudo‐*F*_1,127_ = 3.95, p (perm) = 0.047) while this interaction was not significant for the other variables (ventilation rate: pseudo‐*F*_1,127_ = 0.0002, p (perm) = 0.99; proportion of time spent swimming: pseudo‐*F*_1,127_ = 1.07, p (perm) = 0.30, Fig. 3).

## Discussion

To our knowledge, this is the first study to focus on the evolutionary aspects of pipefish reproductive ecology in several species regarding (1) how embryo size and survival and brooding males' behavior are affected by ambient hypoxia, (2) how the effects of hypoxia are further impacted by the presence or absence of the brood pouch, and (3) how egg size affects embryo survival, size, and male behavior during brooding under hypoxic conditions across species. These areas are discussed below.

### Effects of hypoxia versus normoxia

We found that ambient oxygen availability clearly affects reproductive success in pipefishes, as hypoxia reduced both embryo survival and size across the species that were here investigated. However, these detrimental effects differed significantly between species and were somewhat mediated by the presence or absence of the brood pouch and by the size of the eggs produced by each species (discussed below in relation to the brood pouch and egg size). These results are in line with recent measurements that showed that, in *S. typhle,* oxygen levels within the pouch were substantially lower than in the surrounding waters, both in normoxia and in hypoxia (Braga Goncalves et al. 2015a). In terms of behavior, we found that ventilation rates and proportion of time spent in the top half of the water column increased significantly in hypoxia (discussed below in relation to the brood pouch). Proportion of time spent swimming, however, was unaffected by oxygen conditions. This result may be a product of the general syngnathid behavior, as these fishes rely on crypsis and are thus not active swimmers (Vincent et al. 1995).

### Effects of presence versus absence of brood pouch

The species with brood pouches used in this study showed lower average embryo survival than species lacking brood pouches, and were as affected by hypoxia as were pouchless species. We expected the heavily vascularized brood pouches (Carcupino et al. 2002; Ripley et al. 2010) to be partially an adaptation to supply oxygen to the enclosed embryos, and possibly that the species with brood pouches would do better than pouchless species in hypoxic conditions, but our results show that this is clearly not the case. This result may be generated in two ways. First, in this study, the species with brood pouches also include the species that produced the largest eggs. It is thus difficult to disentangle the effects of egg size from those of the brood pouch, in particular as *S. rostellatus* (with a pouch and small eggs) showed intermediate embryo survival, but also because we lack a species representing the large eggs and no pouch combination. Seadragons (genus *Phyllopteryx* or *Phycodurus*) would have been such examples, but as they only occur in West and Southern Australia under different ecological conditions, it was not tenable to include them in this study. Second, in addition to all the potential advantages brood pouches provide in terms of care (e.g., physical protection, nutrient provision, and osmoregulation) to the developing embryos, brood pouches may also enable males to have greater control over the number of embryos brooded and which embryos to support (Paczolt and Jones 2010; Sagebakken et al. 2010). Thus, the generally lower embryo survival found in the species with brood pouches indicates that pouches did not evolve primarily to improve offspring survival, but more likely as a response to other selective processes such as mate choice (Paczolt and Jones 2010) or family conflicts (Kamel et al. 2010).

As predicted, pouchless species spent significantly more time close to the water surface, where gas exchange between air and water naturally occurs, and is presumably richer in oxygen compared to that lower in the water column. By spending more time closer to the water surface, males lacking brood pouches may keep themselves and their eggs surrounded by oxygen‐richer water, a feat that would be less useful to males that have their eggs enclosed within a brood pouch. In nature, however, spending more time close to the surface may be costly, especially as brooding *N. ophidion* males face increased predation risk compared to females and nonbrooding males, possibly due to the bright yellow eggs of this species (Svensson 1988). The increase in time spent in the upper part of the water column was not reflected in more active swimming by these species, but rather the opposite. In addition, species with brood pouches had lower ventilation rates, also contrary to what might have been expected given their higher swimming activity and the fact that oxygen supply to the embryos is performed via the paternal blood stream.

### Effects of egg size

In fishes, large egg sizes have been repeatedly linked to larger offspring sizes, which in turn typically have higher growth, increased survival, or other fitness benefits (Bagenal 1969; Ahnesjö 1992; Chambers 1997; Heath and Blouw 1998; Einum and Fleming 1999). Here, we found a clear negative effect of egg size on embryo survival, with large eggs (i.e., *S. typhle* l and *S. typhle* s) presenting the lowest levels of offspring survivorship. Therefore, it is possible that the size advantage reported in other studies is only significant once embryos have hatched and are no longer constrained by the egg membrane to access oxygen (Balon 1984; Sargent et al. 1987). On the one hand, the large egg size species had the heaviest embryos, but they also suffered the greatest reduction in embryo weight in hypoxic compared to normoxic conditions. As conversion efficiency of energy stores into somatic growth is dependent on oxygen availability (Hamor and Garside 1977; Rombough 1988; Diez and Davenport 1990), it is perhaps not surprising that the strongest effect was found in the species with the largest embryos. On the other hand, in a within‐species comparison on *S. typhle*, specifically analyzing survival of embryos originating from relatively small and large eggs in hypoxia and in normoxia, we found no effect of egg size (Braga Goncalves et al. 2015b). This means the negative effects of large egg size, suggested from this present study, only appear on the between‐species level with larger differences in egg size.

Egg size is often associated with maternal traits, such as body size and age, and can itself be under direct and indirect selection for a multitude of reasons resulting in complex life‐history puzzles (Rollinson and Rowe 2015 and references therein). In syngnathids in particular, larger eggs may be selected due to: (1) male mate choice and female competition (Berglund et al. 1986b, 2005; Berglund 1991), resulting in larger females (which in some species produce larger eggs) having higher reproductive success; (2) coevolution with paternal care, for instance, if level of care is adjusted according to egg size, and (3) potential coevolution with brain size, as positive associations between egg size and brain size have recently been found in a phylogenetic comparison (Tsuboi 2015).

### The snake pipefish, *Entelurus aequoreus*

The longest species in this study, the snake pipefish, *E. aequoreus,* stands out from the other species in several ways. Despite its very small eggs, after a brooding period of 18 days, *E. aequoreus* had some of the longest embryos measured and the most striking decrease in embryo survival as well as in embryo length when kept in hypoxic conditions. Moreover, despite the long embryos, these were very thin, with average embryo dry weights being similar to those of *N. ophidion* and *S. rostellatus*. As respiration at the embryonic stage is mostly carried out through the skin, a very thin and elongated body shape as that of *E. aequoreus* embryos may present an adaptation that enables embryos to have higher metabolic and developmental rates than those of the other sympatric species. Although not formally measured, the *E. aequoreus* embryos appeared to be considerably more developed after 18 days of brooding than the embryos of the other species (I. Braga Goncalves, personal observation). Such faster developmental rates may explain why *E. aequoreus* embryos are less tolerant to decreases in ambient oxygen concentrations. Fast developmental rates may also have facilitated the reported population explosion and significant range expansion that took place in the northeastern Atlantic between 2004 and 2006 (Fleischer et al. 2007; Harris et al. 2007).

### General conclusions and evolutionary patterns

Several clear patterns have emerged from our study. First, brooding for 18 days in hypoxia caused a significant decrease in embryo survivorship across species, leading us to question the extent to which brood pouches protect embryos from hypoxic conditions and also to question how much oxygen males are able to transfer to the developing offspring. A previous study on *S. typhle* (Braga Goncalves et al. 2015a) showed that pouch fluid oxygen levels were substantially lower than those of the surrounding water, both in normoxia and hypoxia. Thus, it is possible that the ability to oxygenate the embryos in the brood pouch is traded off against the benefits of other care capabilities, such as better physical protection, provision of nutrients, and osmoregulation.

Second, species with and without brood pouches displayed different behavioral adaptations to hypoxia. Pouchless species that brood the embryos exposed to the ambient water spent significantly more time close to the water surface indicating some degree of behavioral flexibility by moving to somewhat better oxygenated waters.

Third, as expected, egg size had an overall significant positive effect on embryo length and weight, with *S. typhle* producing some of the longest and heaviest embryos. In hypoxia, however, embryos from large eggs suffered greater reductions in survivorship and size, results that were detected between species, but not when exploring the smaller within‐species variation in egg size in *S. typhle* (Braga Goncalves et al. 2015b).

In conclusion, the brooding structures of syngnathids are thought to have evolved to reduce embryo mortality (Wilson et al. 2001) and to enable greater quality of care, including oxygenation (Kolm and Ahnesjö 2005; Kvarnemo 2006; Stölting and Wilson 2007). Yet, the role of brood pouches as a well‐developed paternal oxygenation structure is put into question in our study, as we found pouch brooding to be costly in terms of embryo survival and growth, especially when brooded in hypoxia. This result advances our understanding of the costs and benefits that may have contributed to paternal care evolution in general, and to brood pouch evolution in the family Syngnathidae, in particular. The intriguing question whether access to oxygen is a major constraining factor in the evolution of egg size in aquatic environments, however, remains open.

## Conflict of Interest

None declared.

## Supporting information


**Figure S1.** Ventral side of a recently mated (A) *Nerophis ophidion* and (B) *Entelurus aequoreus* male showing the trunk covered with eggs.
**Table S1.** Male and female body sizes (mm, SL) given as mean ± SE (min–max).
