# Supplementary material for: Evolutionary ecology of pipefish brooding structures: embryo survival and growth do not improve with a pouch
Source: Ecol Evol. 2016 Apr 24;6(11):3608–20. doi: 10.1002/ece3.2139 (PMC4864203; doi:10.1002/ece3.2139)
Supplement: Supplementary file 1 — Figure S1. Ventral side of a recently mated (A) Nerophis ophidion and (B) Entelurus aequoreus male showing the trunk covered with eggs. Table S1. Male and female body sizes (mm, SL) given as mean ± SE (min–max). [file ECE3-6-3608-s001.docx]

**Supplementary Table 1.** Male and female body sizes (mm, SL) given as mean ± SE (min-max).

| **Species** | **Oxygen** | **Male** |  |  |  | **Female** |
| --- | --- | --- | --- | --- | --- | --- |
|  | **treatment** | **size** | **t-test** | | | **size** |
|  |  |  | t | df | p |  |
| *S. rostellatus* | high | 112.9±1.8 (98-126) | 0.45 | 30 | 0.65 | 90-137 |
|  | low | 111.9±1.5 (102-120) |  |  |  |  |
| *S. typhle s* | high | 171.9±1.9 (155-180) | 0.54 | 23 | 0.96 | 160-180 |
|  | low | 172±0.9 (165-175) |  |  |  |  |
| *S. typhle l* | high | 169.4±7.2 (165-180) | 0.66 | 21 | 0.52 | 230-260 |
|  | low | 167.7±5.2 (160-174) |  |  |  |  |
| *N. ophidion* | low | 172±0.9 (165-175) | 1.49 | 37 | 0.14 | 180-292 |
|  | low | 179.8±3.0 (161-217) |  |  |  |  |
| *E. aequoreus* | high | 391.4±3.8 (254-308) | 1.7 | 24 | 0.1 | 277-458 |
|  | low | 307.6 ± 9.5 (250-386) |  |  |  |  |

**Supplementary figure 1.** Ventral side of a recently mated a) *Nerophis ophidion* and b) *Entelurus aequoreus* male showing the trunk covered with eggs.

**
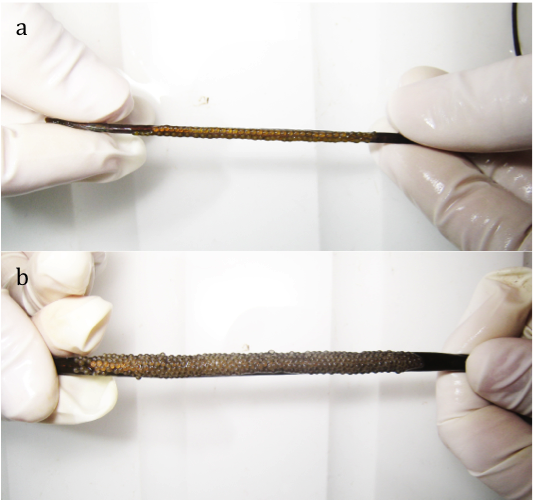
**
